# Prosthetic Valve Endocarditis and Aortic Root Abscess: A Case of High-Risk Infection

**DOI:** 10.7759/cureus.82063

**Published:** 2025-04-11

**Authors:** Alisha Imran, Andrew Quarrell, Leyan Edhem, Gedoni Eni, Adnan Ahmed, Jhiamluka Solano

**Affiliations:** 1 Cardiology, Northern Lincolnshire and Goole NHS Foundation Trust, Grimsby, GBR; 2 General Surgery, Scunthorpe General Hospital, Scunthorpe, GBR; 3 Cardiology, Scunthorpe General Hospital, Scunthorpe, GBR; 4 Internal Medicine, Scunthorpe General Hospital, Scunthorpe, GBR; 5 Cardiology, Castle Hill Hospital, Cottingham, GBR; 6 Resident Doctor Committee, Royal College of Physicians, London, GBR; 7 Education Committee, Academy of Medical Educators, Cardiff, GBR

**Keywords:** aortic root abscess, cardiac pet scan, ct coronary angiography, infective endocarditis, multimodal imaging, prosthetic valve endocarditis

## Abstract

Aortic root abscess is a severe complication of infective endocarditis (IE), particularly in patients with prosthetic valves, nearly doubling mortality risk. Due to the potential for rupture and systemic spread, urgent surgical intervention is recommended. We present the case of a 77-year-old man with a recent aortic valve replacement and a history of discitis who presented with non-specific symptoms, complete heart block, and persistent *Staphylococcus epidermidis* bacteremia. Multimodal imaging, including positron emission tomography (PET) and CT coronary angiography, confirmed an aortic root abscess. Following the IE multidisciplinary team (MDT) recommendations, the patient underwent successful aortic valve and root repair. Post-operatively, he required a pacemaker and dual antibiotic therapy for eight weeks, with no further infection detected. This case highlights the diagnostic challenges of prosthetic valve endocarditis, the critical role of multimodal imaging in detecting complications, and the necessity of early surgical intervention. The development of a heart block underscores the impact of aortic root abscess on conduction pathways. MDT management was essential in optimising patient outcomes.

## Introduction

The formation of an aortic root abscess is one of the most severe complications of infective endocarditis (IE) [1]. Over the past two decades, the incidence of IE in Europe has doubled [2]. The presence of a prosthetic valve significantly increases the risk of developing IE, accounting for approximately 30% of all cases. Notably, the risk of IE increases in the first two years following prosthetic valve replacement, with peak incidence between six months and two years [3]. In addition to the increased risk of developing IE, prosthetic valves also predispose patients to complications, most notably aortic root abscesses. Whilst the overall mortality rate in uncomplicated IE is around 25%, the presence of an aortic root abscess increases this figure to 40% [3]. This increased risk of mortality is primarily due to the susceptibility of an aortic root abscess to rupture and spread within the aorta and surrounding structures [1,3]. Hence, radical treatment through surgical intervention like valve re-replacement, debridement of infected tissue and aortic root reconstruction is recommended in such cases to reduce morbidity and mortality [1].

## Case presentation

**Figure 1:**
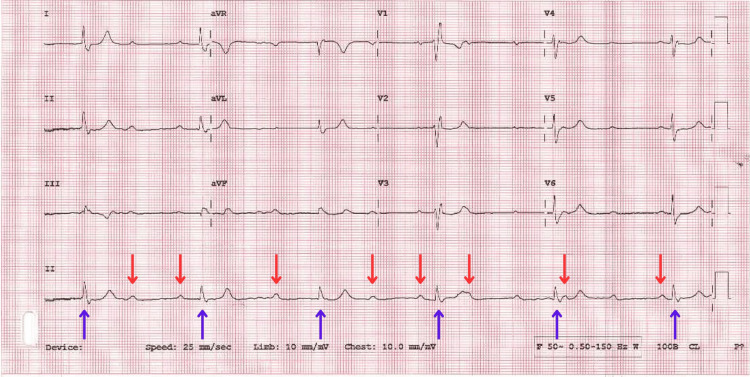
ECG showing third-degree heart block. Complete heart block (third-degree AVB) as evidenced by the atria (red arrows) and ventricular (blue arrows) dissociation. Some missing P waves are hidden or superimposed in the QRS or T wave as noted after the fourth QRS (T wave with superimposed P wave). AVB: atrioventricular block

In 2024, a 77-year-old male presented to the emergency department (ED) following a fall preceded by a two-week history of feeling generally unwell, muscle weakness, and vomiting. He had a background of chronic kidney disease stage 3, osteoarthritis, spondylosis, pulmonary hypertension, aortic valve replacement (AVR) in 2023, coronary heart disease, type 2 diabetes, gout, hypercholesterolemia, bilateral cataracts and a recent diagnosis of discitis. An ECG was performed prior to his arrival, which showed a complete heart block and a new oxygen requirement (Figure 1).

**Figure 2:**
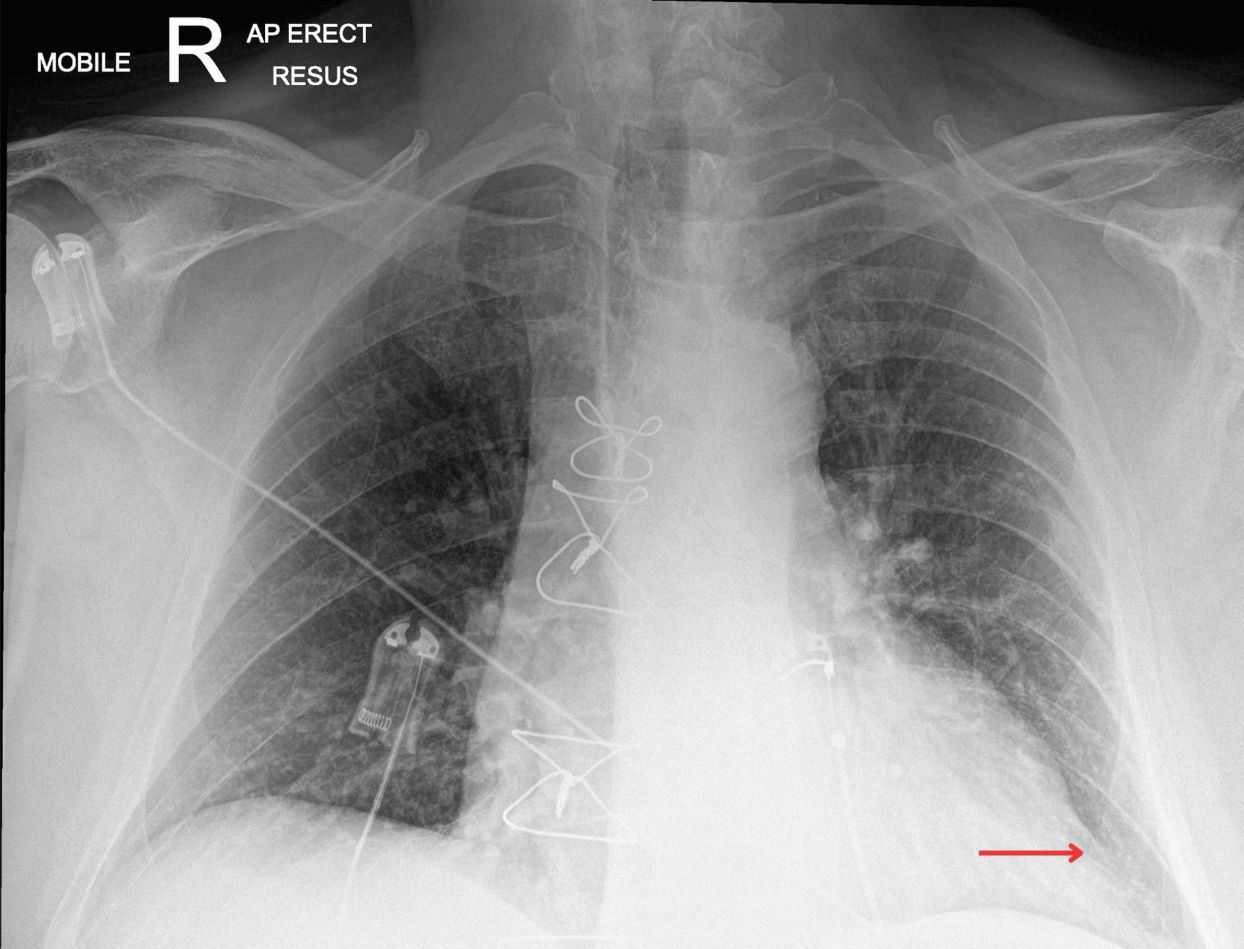
Admission chest X-ray. The arrow showing the area of suspected consolidation.

On arrival to the ED, the patient was bradycardic at 35 bpm and alert. Due to the severe bradycardia, the patient was taken straight to the resuscitation area. Initial observations revealed an oxygen saturation of 96%, requiring 2L of oxygen, a respiratory rate of 19, a blood pressure of 90/52 and a temperature of 39.3°C. Laboratory tests revealed elevated inflammatory and infection markers, with deranged electrolytes, acute kidney injury (AKI) stage 2 and mildly deranged liver function tests (Table 1). A set of blood cultures were taken from the peripheral vein, growing gram-positive *Staphylococcus epidermidis*. A chest X-ray at the time showed possible left lower zone consolidation. The patient was commenced on empirical antibiotic therapy for suspected sepsis (see Figure 2).

**Table 1:** Initial investigations performed on admission. ALT: alanine transaminase; ALP: alkaline phosphatase

Investigations	Patient value	Normal range
White blood cells	18.9	4.0-11 x 10^9^/L
C-reactive protein	145	0-8 mg/L
Neutrophils	17.29	2-7.7 x 10^9^/L
Urea	15.4	3.0-7.6 mmol/L
Creatinine	202	65-114 µmol/L
Potassium	5.6	3.5-5.3 mmol/L
ALT	60	5-45 U/L
ALP	153	30-125 U/L
Bilirubin	25	<21 µmol/L

Whilst in the resuscitation area, the patient’s cardiac monitoring started showing long pauses, and the heart rate reduced to <10 bpm. The Advanced Life Support algorithm was followed, an isoprenaline infusion was initiated following the cardiologist's advice, and a temporary pacemaker was inserted. A bedside transthoracic echocardiogram (TTE) revealed a left ventricular ejection fraction (LVEF) greater than 65%, poor right ventricular (RV) function, a dilated right atrium (RA), normal valves, and AVR (see Video 1). After initial resuscitation and once the patient was stable, he underwent a transesophageal echocardiogram (TOE), and his case was further discussed at the mitral valve and endocarditis multidisciplinary team (MDT) meeting.

**Video 1:** Transesophageal echocardiogram (TOE) and transthoracic echocardiogram (TTE). A: TOE aortic valve long-axis view; B: TOE aortic valve long-axis zoomed view; C: TOE aortic valve long-axis 3D view; D: TTE apical 5 chambers view

**Figure 3:**
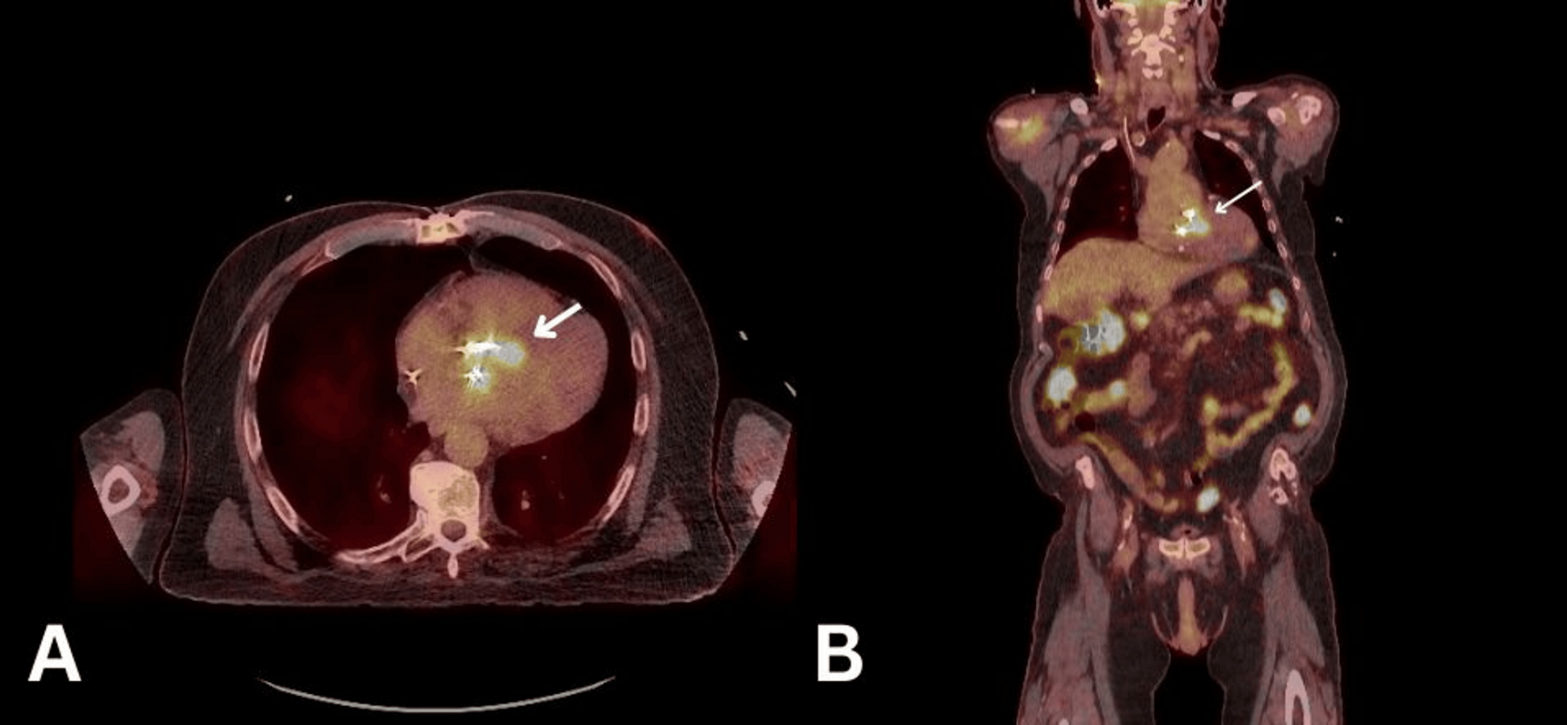
PET scan showing intense activity was seen throughout the aortic valve replacement (white arrow). A: transversal view; B: coronal view; PET: positron emission tomography

**Figure 4:**
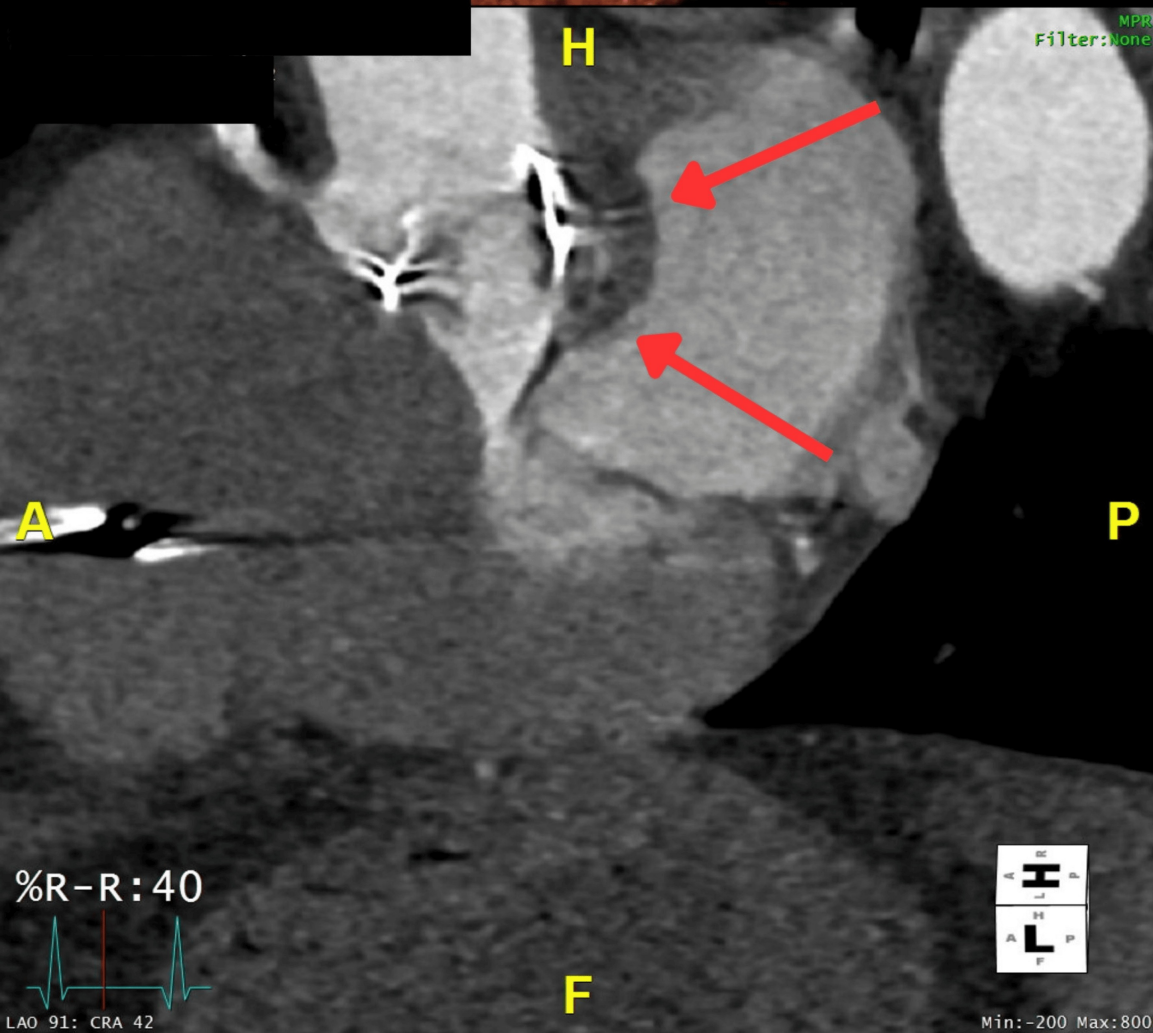
CT cardiac coronary angiogram showing aortic root abscess (red arrows).

Considering that the patient was recently diagnosed with multi-level discitis, another recurrence of infection was considered. A whole-body positron emission tomography (PET) scan was performed (see Figure 3), and antibiotics were changed to daptomycin and rifampicin following the microbiologist's advice. The PET scan revealed several concerning findings: intense activity was seen throughout the AVR. In addition, several sites of concern, including the thoracolumbar spine, diffuse, homogenous activity in relation to AVR and external pacing wire, could all represent ongoing infection. Given the findings of the PET scan, it was advised to perform a CT cardiac coronary angiogram to rule out an aortic root abscess. The angiogram revealed that there appeared to be soft tissue noted adjacent to the non-coronary cusp and the interatrial septum, measuring approximately 1.2 cm. In the delayed phase, there was some minor contrast opacification centrally. This result corresponded with the abnormality seen on the TOE, which was consistent with the presence of an aortic root abscess (see Figure 4).

The patient’s results were discussed in a follow-up IE MDT meeting, which concluded that the patient had IE of the prosthetic valve and an aortic root abscess formation, thus requiring urgent surgical intervention. The patient underwent a bioprosthetic AVR repair plus aortic root repair.

Post-operatively, the patient was hemodynamically stable and transferred to the Intensive Care Unit (ICU) due to the nature of the operation, where they resided for seven days. ICU complications included pneumonia and a worsening right-sided effusion requiring chest drain insertion. In addition, the patient required a new pacemaker fitted due to a persistent complete heart block despite having a temporary pacemaker fitted. Additionally, the patient suffered a few episodes of atrial fibrillation, which prompted the decision to escalate the temporary wire to a single-chamber VVVI pacemaker rather than a dual-chamber one.

A repeat transthoracic echocardiography (TTE) was performed two weeks post-AVR to assess the left ventricle (LV) function. The results concluded that the LV systolic function appeared well preserved and that the aortic root appeared dilated, with the valve having a peak gradient of 20.4 mmHg. A post-op infectious disease MDT meeting was also held, and it was concluded that no growth was found on the aortic valve post-operatively. The patient was initiated on a regimen of doxycycline and dalbavancin for eight weeks. Following this, the patient was discharged and subsequently followed up in the pacing clinic at two weeks and in the outpatient IE (OP IE) clinic six to eight weeks post-discharge, with continued care provided through the Outpatient Parenteral Antimicrobial Therapy (OPAT) service.

## Discussion

The development of aortic root abscess is a serious complication of IE, with recent meta-analysis data showing increased mortality both in the hospital and following discharge in the later stages [4]. Prosthetic valve replacement is a crucial factor for the development of aortic root abscesses [5], and our patient presented within a high-risk period of around two years post-operatively [6]. A prompt medical/microbiological diagnosis, radiological confirmation and urgent surgical revision are essential to ensure the best chances of survival in cases of aortic root abscess [7].

In cases of IE, perivalvular abscesses most commonly develop in the aortic region [8], with up to 46% of cases of infective native aortic valve endocarditis resulting in root complications [9]. Constitutional changes to the native valve also act as risk factors, including congenital bicuspid valves and, in our case, a prosthetic valve replacement [5,10]. The clinical presentation of our patient included constitutional symptoms of malaise and fever, in addition to bradycardia and a complete heart block. Mahmoud et al. described key predictors of aortic root abscess, with constitutional symptoms, heart failure and conduction abnormalities as indicators of abscess over uncomplicated IE [11]. PR prolongation is the most common ECG abnormality seen in aortic root abscess. Abscess can erode into the conduction pathways near the aortic root (e.g., the atrioventricular node and His-Purkinje system), leading to heart block. In prosthetic valve settings, fibrosis or scarring from the valve replacement may predispose to such conduction disturbances [12]. Our patient was shown to have complete heart block and severe bradycardia. A previous case study showed early mortality in aortic root abscess with complete heart block, indicating our patient had severe electrophysiological changes as a result of their illness [13]. To add to the poor prognostic ECG changes, the patient in this study was found to have AKI stage 2 on laboratory investigations. Renal impairment is shown to be a significant determinant of one-year mortality in patients with perivalvular abscess [14].

An untreated aortic root abscess can cause serious complications, including obstruction to coronary blood flow and heart block. Thus, prompt management is essential to reduce morbidity and mortality. The involvement of the endocarditis team in this case reflects the current European Society of Cardiology (ESC) guidelines for IE. Due to the diverse presentations of IE, a multidisciplinary approach is recommended to optimise patient outcomes. The endocarditis team should comprise cardiologists, cardiovascular surgeons, infectious disease specialists and microbiologists [15]. In this case, microbiologists played a key role in guiding targeted antibiotic therapy, selecting dalbavancin due to its prolonged half-life, which facilitates less frequent dosing, and its activity against the identified gram-positive pathogen, *S. epidermidis*. Additionally, early surgical intervention was undertaken following the detection of an aortic root abscess, a recognised Class I, Level A indication recommended for patients with prosthetic valve endocarditis and aortic root abscess [15].

Evidence has shown that early surgical intervention for complicated IE cases has the potential to improve one-year survival rates by 15-20% [15]. Early surgical intervention in complicated IE is necessary as antibiotics alone are insufficient in eradicating infectious organisms, with a high risk of significant tissue damage if surgery is delayed [16]. When surgery is indicated, further assessment of coronary anatomy is recommended, and pre-operative coronary angiography is recommended in men >40 years old. This recommendation was executed in our case, with the patient undergoing a pre-operative coronary angiography. Although in this case, pre-operative coronary angiography was performed, it is worth noting that the presence of aortic valve vegetations may prevent invasive imaging, and alternative non-invasive imaging may be preferable to avoid procedural risks, including iatrogenic embolisation. In our case, the decision to proceed with invasive imaging was carefully weighed against these risks. Alongside imaging, pre-operative antibiotic therapy must be continued intra-operatively, with dose adjustments in prolonged procedures or cases of significant blood loss [15]. The choice of surgical intervention in cases of complicated IE has been widely researched, with current literature suggesting that all surgery should include thorough debridement of diseased and necrotic tissue around the aortic root [16]. In addition to debridement, aortic root reconstruction by patching and AVR, as observed in our case, is a commonly preferred technique [16]. In this case, no growth was found on the aortic valve post-operatively; this favourable outcome following early surgery aligns with evidence demonstrating improved survival rates in early surgical interventions in complicated IE cases.

## Conclusions

This case highlights the critical nature of aortic root abscess as a life-threatening complication of IE, particularly in patients with prosthetic valves. Our patient's presentation with conduction abnormalities and sepsis underscores the importance of early recognition and a multidisciplinary approach in managing such high-risk cases. Prompt microbiological diagnosis, advanced imaging, and urgent surgical intervention played a pivotal role in optimising outcomes, aligning with current ESC guidelines. The successful resolution of infection post-operatively reinforces the value of early surgical debridement and targeted antimicrobial therapy in improving survival and reducing morbidity in complicated IE cases.
